# Preparation of CsPb(Cl/Br)_3_/TiO_2_:Eu^3+^ composites for white light emitting diodes

**DOI:** 10.3389/fchem.2023.1199863

**Published:** 2023-05-19

**Authors:** Chen Zhang, Minqiang Wang, Jindou Shi, Junnan Wang, Zheyuan Da, Yun Zhou, Youlong Xu, Nikolai V. Gaponenko, Arshad Saleem Bhatti

**Affiliations:** ^1^ Electronic Materials Research Laboratory, Key Laboratory of the Ministry of Education International Center for Dielectric Research and Shannxi Engineering Research Center of Advanced Energy Materials and Devices, Xi’an Jiaotong University, Xi’an, China; ^2^ Belarusian State University of Informatics and Radioelectronics, Minsk, Belarus; ^3^ Centre for Micro and Nano Devices, Department of Physics, COMSATS Institute of Information Technology, Islamabad, Pakistan

**Keywords:** CsPb(Cl/Br)_3_/TiO_2_:Eu^3+^, perovskite, anatase, nano-materials, white light emitting diodes

## Abstract

The inherent single narrow emission peak and fast anion exchange process of cesium lead halide perovskite CsPbX_3_ (X = Cl, Br, I) nanocrystals severely limited its application in white light-emitting diodes. Previous studies have shown that composite structures can passivate surface defects of NCs and improve the stability of perovskite materials, but complex post-treatment processes commonly lead to dissolution of NCs. In this study, CsPb(Cl/Br)_3_ NCs was in-situ grown in TiO_2_ hollow shells doped with Eu^3+^ ions by a modified thermal injection method to prepare CsPb(Cl/Br)_3_/TiO_2_:Eu^3+^ composites with direct excitation of white light without additional treatment. Among them, the well-crystalline TiO_2_ shells acted as both a substrate for the dopant, avoiding the direct doping of Eu^3+^ into the interior of NCs to affect the crystal structure of the perovskite materials, and also as a protection layer to isolate the contact between PL quenching molecules and NCs, which significantly improves the stability. Further, the WLED prepared using the composites had bright white light emission, luminous efficiency of 87.39 lm/W, and long-time operating stability, which provided new options for the development of perovskite devices.

## 1 Introduction

Cesium lead halide perovskite CsPbX_3_ (X = Cl, Br, I) nanocrystals (NCs) has attracted a lot of attention from researchers due to its excellent optoelectronic properties and versatile surface chemistry (Tang et al., 2016; Zhou et al., 2016; Pan et al., 2018a). White light-emitting diodes (WLEDs) prepared by CsPbX_3_ NCs have high luminous efficiency and low energy consumption, making them one of the favorable candidates for next-generation photoelectric devices (Dai et al., 2014; Pan et al., 2016; Yang et al., 2019). However, if the light-emitting layer of WLED were to use both CsPbI_3_, CsPbBr_3_ and CsPbCl_3_ as RGB light sources would inevitably lead to impure chromaticity due to anion exchange between halogenated elements (Zhang et al., 2017; Shi et al., 2022). Meanwhile, the problem of instability of CsPbX_3_ NCs in working environment also impeded its further development in the fields of display and lighting (Yang et al., 2015; Shi et al., 2019; Shi et al., 2020; He et al., 2022).

Currently, the mainstream method of combining white light by CsPbX_3_ NCs was achieved by mixing NCs with other phosphors or doping with other luminophor. For example, Yuan et al. sensitized CsPbI_3_ NCs by doping with Yb^3+^ ions, mixed them with Y_3_Al_5_O_12_:Ce^3+^ green phosphors, and integrated them on GaN blue LED chips to finally prepare WLED with good stability (Yuan et al., 2020). Pan et al. (2017) prepared CsPbCl_3_ NCs with Ln^3+^ ion (Ce^3+^、Sm^3+^、Eu^3+^、Dy^3+^、Er^3+^, and Yb^3+^) emission peaks by doping Ln^3+^ ions into the lattice of CsPbCl_3_ NCs. In a subsequent study, they further doped the CsPb(Cl/Br)_3_ NCs with lanthanide ion pairs (Ce^3+^/Mn^2+^、Ce^3+^/Eu^3+^, and Ce^3+^/Sm^3+^) to achieve white phosphors with 75% high photoluminescence quantum yields (PLQY) (Pan et al., 2018b). However, due to the environmentally sensitive nature of CsPbX_3_ NCs, the low efficiency of the prepared LEDs and the instability of the phosphors in each layer were still to be solved (Bae et al., 2013; Fakharuddin et al., 2019). Meanwhile, when the doping ions entered the interior of the CsPbX_3_ NCs lattice, it inevitably influenced the crystal structure of CsPbX_3_ NCs, resulting in the shift of the NCs luminescence wavelength (Huang et al., 2022). Therefore, it is necessary to develop highly efficient and stable composite materials that can directly excite white light.

In this paper, we propose a convenient synthetic strategy to prepare CsPb(Cl/Br)_3_/TiO_2_:Eu^3+^ composites which can directly excite white light. The pre-prepared TiO_2_ hollow shells were placed in the precursor solution and CsPb(Cl/Br)_3_ NCs were in-situ grown inside the shells by thermal injection method. Among it, the pre-prepared TiO_2_ hollow shells acted as hosts for Eu^3+^ ions with bright red light emission (∼614 nm), which both avoided the change of the perovskite crystal structure caused by direct doping and replaced the CsPbI_3_ NCs, solving the problem of anion exchange due to the introduction of I^−^. Further, the stability of the NCs was significantly improved benefited from the protection of the external TiO_2_ hollow shells. The WLEDs prepared using CsPb(Cl/Br)_3_/TiO_2_:Eu^3+^ composites exhibited a luminous efficiency of 87.39 lm/W and long-time operating stability, which greatly enhances the potential competitiveness of perovskite materials for commercial lighting device applications.

## 2 Materials and methods

### 2.1 Materials

The cesium carbonate (Cs_2_CO_3_, 99.99%), lead (II) bromide (PbBr_2_, 99.99%), lead (II) chloride (PbCl_2_, 99.99%), oleic acid (OA, 85%), oleylamine (OAm, 80–90%), 1-octadecene (ODE, 90%), Tetrabutyl titanate (TBOT, >99%) and Tetraethyl orthosilicate (TEOS, >99%) were purchased from Aladdin. Europium (Ⅲ) nitrate hexahydrate (Eu(NO_3_)_3_^**.**^6H_2_O, 99.9%) and isopropyl alcohol (IPA, Analytical Reagent) were purchased from Macklin. The poly (styrene) (PS), ethanol absolute (Analytical Reagent), ammonia solution (NH_4_OH) and toluene (>99.5%) purchased from Shanghai Chemical Industrial Company. The 365 nm UV-chips (10W) and commercial WLED (10W) were purchased from Shenzhen Youjing Optoelectronics factory. All the reagents were used without further purification.

### 2.2 Synthesis of TiO_2_:Eu^3+^ hollow shells

5 mL TEOS was slowly added to the mixture of 0.35 mL NH_4_OH, 2.5 mL deionized water and 20 mL IPA and stirred continuously at room temperature for 5 h to obtain SiO_2_ by hydrolysis. After that, the crude product was centrifuged at 3,000 rpm/min for 5 min to obtain white precipitates, washed several times with deionized water, and dried at 60°C for 12 h to obtain SiO_2_ spherical templates with average particle size of 340 nm.

0.25 g SiO_2_ spherical templates and 0.2–1.2 mmol europium acetate were placed into the mixed solution of 0.75 mL deionized water and 37.5 mL ethanol, and sonicated for 20 min to make them completely dispersed. After that, 1.25 mL TBOT were slowly dropped into the mixed solution and stirred for 5 h at room temperature to make it completely hydrolyzed. The products were annealed at 800°C for 3 h to obtain SiO_2_/TiO_2_:Eu^3+^ composites (The heating rate was 100°C/h). Finally, the composites were etched in ammonia at concentration of 4 M for 14 h until the SiO_2_ spheres disappeared completely, and the secondary annealing was performed under the same conditions to obtain TiO_2_:Eu^3+^ hollow shells.

### 2.3 Synthesis of the CsPb(Cl/Br)_3_/TiO_2_:Eu^3+^ composites

0.814 g Cs_2_CO_3_ were placed in the mixture of 2.5 mL OA and 10 mL ODE and transferred to a 100 mL three-neck flask. The solution was heated to 120 °C under vacuum for 1 h. After that, the solution was heated to 140 °C under N_2_ for another 1 h to obtain the Cs-OA precursor. Cs-OA precursors required to be held at 100 °C before use.

0.1 mmol of PbCl_2_ and PbBr_2_ (PbCl_2_:PbBr_2_ was 9:1, 2:1, 1:1, 1:2 and 1:9, respectively), 0.3 g TiO_2_:Eu^3+^ hollow shells were placed in the mixture consisting of 10 mL ODE, 0.5 mL OA and 0.5 mL OAm and transferred to another 100 mL three-neck flask. After warming to 120 °C for 1 h under N_2_, the reaction was then warmed to 150 °C for 10 min, and then 0.6 mL Cs-OA were injected into the solution. After 5–10 s, the reaction was terminated by immersing the three-necked flask in ice water. The solution was continued to stir at room temperature for 1 h to grow NCs in TiO_2_:Eu^3+^ hollow shells. The crude solution was centrifuged at 2000 rpm/min for 5 min to obtain the precipitate, and washed with toluene 2–3 times. The obtained product was dried under vacuum at 60°C for 5 h to obtain CsPb(Cl/Br)_3_/TiO_2_:Eu^3+^ composites.

### 2.4 Preparation of WLEDs

First, 0.05 g CsPb(Cl/Br)_3_/TiO_2_:Eu^3+^ powders and 0.5 g PS particles were placed in 5 mL toluene and stirred at 60°C for 3 h. Then, the solution was poured into prefabricated molds and CsPb(Cl/Br)_3_/TiO_2_:Eu^3+^ polymer films were deposited at room temperature. Finally, films were coated on a 365 nm UV-chip to obtain LEDs with white light emission.

### 2.5 Characterization

The morphology and microstructure of the samples were analyzed by transmission electron microscope (TEM, JEOL JEM-F200). The energy dispersive spectroscopy (EDS) spectra of the SiO_2_/TiO_2_:Eu^3+^ powder samples were investigated with field emission scanning electron microscope (SEM, FEI Quatan FEG 250) equipped EDS. Photoluminescence (PL) spectra and time-resolved PL (TRPL) decay curves of the samples were recorded on Edinburgh Instruments FLS1000 spectrometer. UV-Vis absorption spectra were recorded with the Jasco V-570 UV/VIS/NIR spectrophotometer. The X-ray diffraction (XRD) patterns were obtained with the DB-ADVANCE X-ray diffractometer. Electroluminescence (EL) spectra of white LEDs were collected by a Keithley 2,400 light source meter and the Photo Research 670 spectrometer.

## 3 Results and discussion

### 3.1 Preparation process of CsPb(Cl/Br)_3_/TiO_2_:Eu^3+^ composites

**FIGURE 1:**
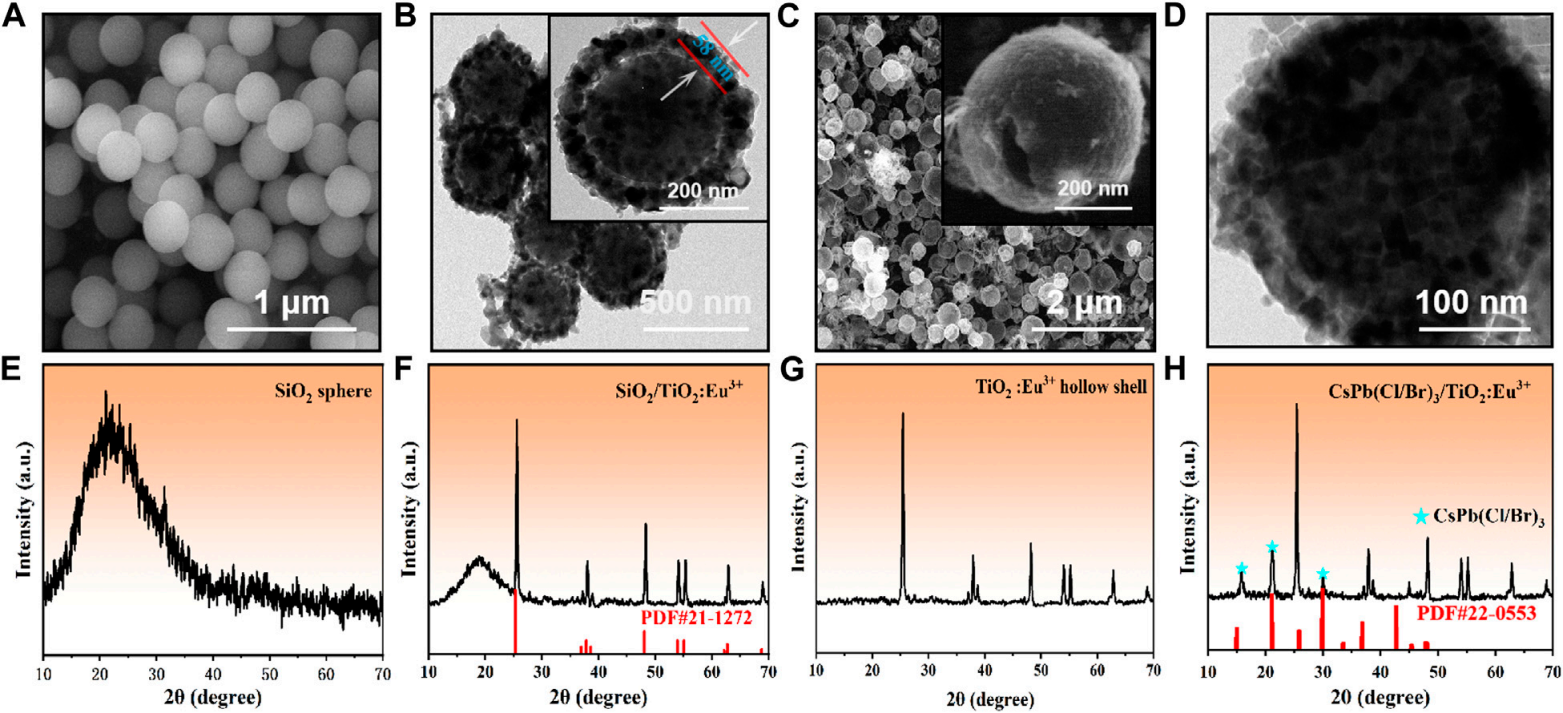
**(A**, **E)** SEM images and XRD patterns of SiO_2_ nanoparticles. **(B**, **F)** TEM images and XRD patterns of SiO_2_/TiO_2_:Eu^3+^ composites. **(C**, **G)** SEM and XRD patterns of TiO_2_:Eu^3+^ hollow shells. **(D**, **H)** TEM images and XRD patterns of CsPb(Cl/Br)_3_/TiO_2_:Eu^3+^ composites. The doping amount of Eu^3+^ ions in this part of the sample was 0.6 mmol, and the annealing temperature was 800°C.

In this paper, CsPb(Cl/Br)_3_/TiO_2_:Eu^3+^ composites with multi-peak emission were obtained by a two-step method (Supplementary Figure S1). First, we synthesized a series of monodisperse SiO_2_ spheres as nanotemplates for the preparation of TiO_2_ hollow shells. The pre-prepared SiO_2_ nanoparticles exhibited uniform size (approximately 340 nm) and smooth surface, which are ideal template materials (Figure 1A; Supplementary Figures S2A, B). Afterwards, SiO_2_/TiO_2_:Eu^3+^ composites were obtained by TBOT hydrolysis and annealed at high temperature. The energy dispersive spectroscopy (EDS) showed that the Ti elements were uniformly distributed on the surface of the SiO_2_ spheres, forming mesoporous TiO_2_ shells (Supplementary Figure S3). The average size was approximately 460 nm, and the shell thickness was approximately 58 nm (Figure 1B; Supplementary Figures S2C, D). The corresponding XRD results showed two characteristic diffraction peaks for amorphous SiO_2_ and anatase TiO_2_ (PDF#21-1272) (Figure 1F), and no other impurity phases appeared, attributed to the low doping concentration of Eu^3+^ ions in the surface TiO_2_ layer. Further, a series of hollow TiO_2_:Eu^3+^ shells were obtained by using ammonia to, etch the SiO_2_ templates (Figure 1C) (Chang et al., 2017). The inset showed the surface pores of the TiO_2_:Eu^3+^ hollow shells, which can ensure the smooth entry and crystallization of Cs^+^, Pb^2+^, Cl^−^ and Br^−^ ions from the solution into the shells. At this time, the XRD pattern also showed that the broad peak of amorphous SiO_2_ had completely disappeared (Figure 1G), which proved that the SiO_2_ template could be effectively removed by prolonged etching with ammonia. The corresponding EDS spectra showed that the Eu^3+^ ions were uniformly distributed in the TiO_2_ hollow shells without agglomeration (Supplementary Figure S4). Finally, CsPb(Cl/Br)_3_ NCs were grown *in situ* in TiO_2_:Eu^3+^ hollow shells to obtain CsPb(Cl/Br)_3_/TiO_2_:Eu^3+^ composites. Most of the CsPb(Cl/Br)_3_ NCs have entered the TiO_2_:Eu^3+^ hollow shells as observed by TEM images (Figure 1D), and the XRD pattern of this materials (Figure 1H) showed the diffraction peaks of CsPb(Cl/Br)_3_ NCs (PDF#22-0553) in addition to the diffraction peaks of anatase phase TiO_2_, which indicated that both had completed the composite. In summary, the formation process of CsPb(Cl/Br)_3_/TiO_2_:Eu^3+^ was demonstrated by SEM, TEM and XRD variations, which confirmed that the composites could be successfully obtained by a simple two-step method.

### 3.2 CsPb(Cl/Br)_3_ and CsPb(Cl/Br)_3_/TiO_2_:Eu^3+^ crystal structures

**FIGURE 2:**
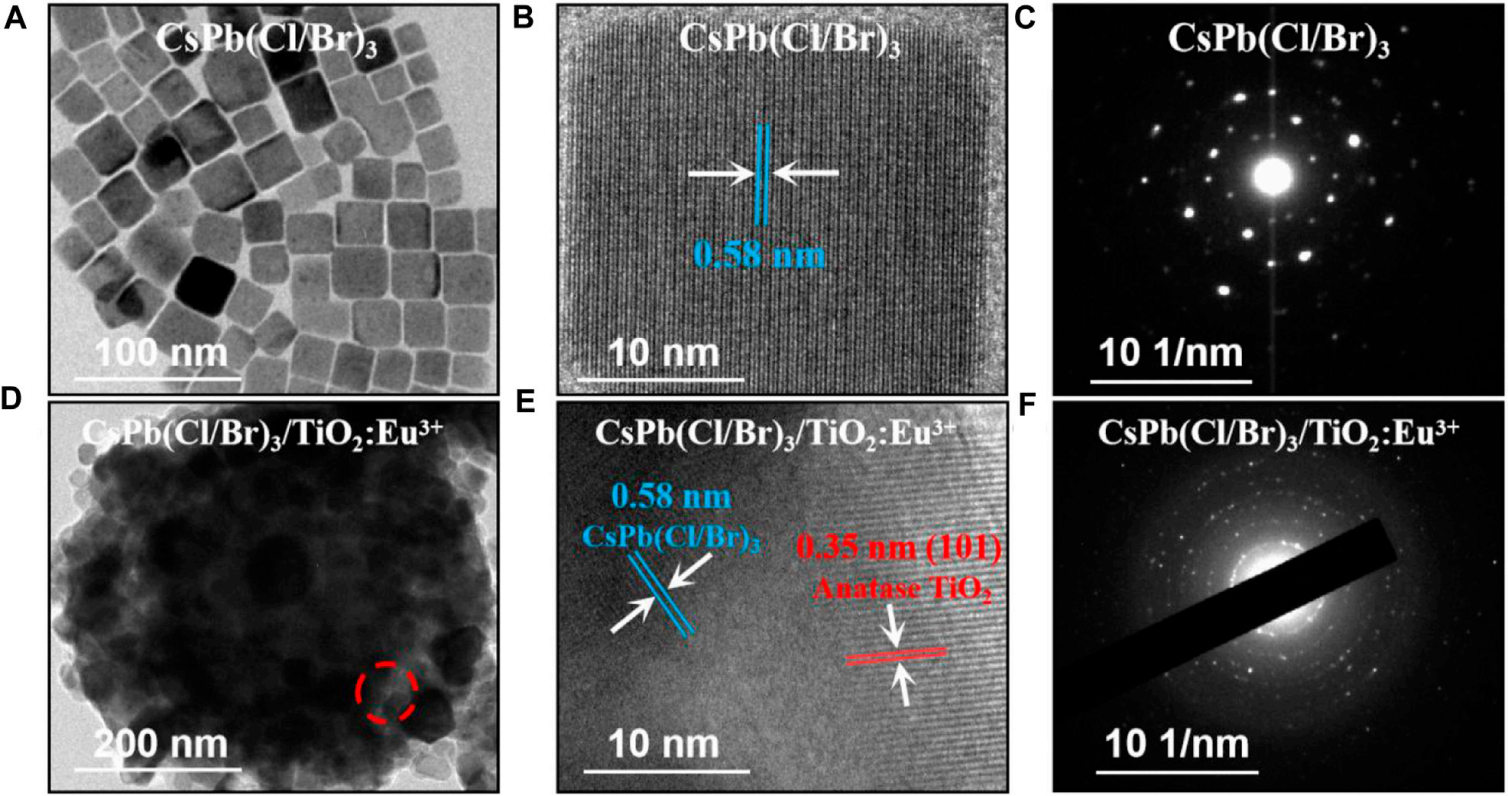
**(A)** TEM, **(B)** HRTEM and **(C)** SAED images of CsPb (Cl/Br)_3_ NCs. **(D)** TEM, **(E)** HRTEM at the position of the red circle and **(F)** SAED images of CsPb (Cl/Br)_3_/TiO_2_:Eu^3+^ composites.

Subsequently, the microscopic morphology and crystal structure of CsPb(Cl/Br)_3_ NCs and CsPb(Cl/Br)_3_/TiO_2_:Eu^3+^ composites were further analyzed. First, the pure CsPb(Cl/Br)_3_ NCs had average size of approximately 26 nm and had good dispersion (Figure 2A; Supplementary Figures S2E, F). The corresponding high-resolution transmission electron microscopy (HRTEM) images showed clear lattice stripes with crystal plane spacing of about 0.58 nm (Figure 2B). Selected area electron diffraction (SAED) images were typical of single-crystal diffraction spots, indicating that the prepared CsPb(Cl/Br)_3_ NCs have good crystallinity (Figure 2C) (Song et al., 2015; Yan et al., 2020). Similarly, the CsPb(Cl/Br)_3_/TiO_2_:Eu^3+^ composites were observed by TEM and many cubic CsPb(Cl/Br)_3_ NCs were found to be encapsulated within TiO_2_:Eu^3+^ hollow shells (Figure 2D). Afterwards, two lattice fringes with different spacing could be clearly observed at the red circle in Figure 2D, corresponding to CsPb(Cl/Br)_3_ NCs (spacing 0.58 nm) and the TiO_2_:Eu^3+^ hollow shells [spacing 0.35 nm, anatase (101) crystalline surface] (Figure 2E). Also, the multilayer ring-like SAED images further verified the excellent crystallinity of the composites (Figure 2F) (Dong et al., 2021).

### 3.3 Optical properties of TiO_2_:Eu^3+^ hollow shells

In order to make the materials emit white light directly, it is necessary to match the fluorescence intensity of different light sources. However, compared with the high quantum yield of CsPbX_3_ NCs, the excitation light of Eu^3+^ ions was often hidden (Wang et al., 2017; Shi et al., 2021; Xu et al., 2022). Therefore, the effects of the concentration of Eu^3+^ ions and annealing temperature were investigated in this paper to make the TiO_2_:Eu^3+^ hollow shells showing the brightest red light emission. First, the TiO_2_ hollow shell samples with different doping amounts were white powders under daylight, but emitted different intensities of red light under 365 nm UV excitation (Supplementary Figure S5A). Clearly, the 0.6 mmol doped sample had the brightest red light effect. The corresponding PL spectra proved this point (Supplementary Figure S5B), and the PL intensity began to decrease with further increase of doping amount. This phenomenon was attributed to the concentration quenching effect, where the central distance between ions gradually decreases as the concentration of Eu^3+^ ions increases, leading to an increase in the cross-relaxation rate, which adversely affects the fluorescence intensity (Gao et al., 2021). Meanwhile, the XRD results indicated that the PL intensity decrease was also related to the phase change of the TiO_2_ host. High concentration (0.8 mmol and above) of Eu^3+^ doping converted anatase TiO_2_ to Eu_2_Ti_2_O_7_ (PDF#23-1072) and rutile TiO_2_ (PDF#21-1276) (Supplementary Figure S5C). However, due to the highly symmetric crystal structure of Eu_2_Ti_2_O_7_, the products had non-PL activity (Mrázek et al., 2015; Orihashi et al., 2016). Thus, the concentration quenching effect and the substrate phase transition both together lead to the decay of the emission intensity of TiO_2_:Eu^3+^ hollow shells above 0.8 mmol. Through Rietveld refinement of XRD data, the content of each phase in the product at this time was calculated to determine the conversion rate of anatase (Supplementary Figure S6). The results showed that 73.2% of the anatase TiO_2_ substrate underwent phase transformation, among which rutile accounted for 33.2% of the total and Eu_2_Ti_2_O_7_ was 40.0%. Therefore, precise control of the Eu^3+^ ion doping ratio was crucial. In addition, the main peaks of both the anatase TiO_2_ and Eu_2_Ti_2_O_7_ were slightly shifted in the small-angle direction as the Eu^3+^ doping in the TiO_2_ host increased, attributed to the replacement of the smaller Ti^4+^ (61 p.m.) by the larger ionic radius Eu^3+^ (94.7 p.m.) (Supplementary Figure S5C) (Pan et al., 2017). Consistent results were also observed in the UV-Vis absorption spectra (Supplementary Figure S5D), confirming that the increase of Eu^3+^ doping would lead to the rapid transformation of anatase TiO_2_ to Eu_2_Ti_2_O_7_. In summary, the 0.6 mmol Eu^3+^ ion doped TiO_2_ has both no impurity generation and the strongest red light emission.

Similarly, the annealing process was an essential step for the formation of highly crystalline TiO_2_:Eu^3+^ hollow shells and played a decisive role in the optical properties of the Eu^3+^ ions (Werts et al., 2002; Reszczyńska et al., 2016). We further investigated the effect of temperature on the fluorescence intensity of TiO_2_:Eu^3+^ hollow shells. The PL spectra showed that the emission intensity of samples peaked at 800°C (Supplementary Figure S7A). The XRD results revealed the reason for the fluorescence change (Supplementary Figure S7B). The diffraction peaks of anatase TiO_2_ gradually became stronger at 600–800°C, especially the sharp diffraction peaks at 800°C indicated that the TiO_2_:Eu^3+^ hollow shells had the best crystallinity at this time. However, when the annealing temperature rises to 900°C, the products showed a transformation similar to the previous results (from anatase to rutile and Eu_2_Ti_2_O_7_). It was concluded that at the 600–800°C range, the increased annealing temperature resulted in a better crystallinity of the TiO_2_ host and lower density of defect states between the grains, leading to stronger fluorescence emission (Ningthoujam et al., 2009; Chang et al., 2017). When the temperature was further increased (900°C), the sub-stable anatase TiO_2_ reacted with Eu^3+^ ions free inside the lattice to form Eu_2_Ti_2_O_7_, leading to a decrease in fluorescence intensity. The XRD results of TiO_2_:Eu^3+^ hollow shells at low temperatures (300–500°C) showed no or very low diffraction peaks, indicating that the lowest temperature to make hollow shells crystallize was 500°C (Supplementary Figure S8). In addition, we also compared the phase transition process of pure TiO_2_ at different annealing temperatures and found that the anatase to rutile transition occurred at 800°C (Supplementary Figures S9A, B). This phenomenon proved that the doping of Eu^3+^ ions could stabilize the crystal structure of anatase TiO_2_ and make it withstand higher temperatures without phase transformation. Subsequently, the optical properties of TiO_2_:Eu^3+^ hollow shells at different annealing temperatures were further investigated. The corresponding UV-Vis absorption spectra showed that the absorption edge of the samples appeared to change significantly when the temperature was increased to 900°C (Supplementary Figure S7C), which was another proof that high temperature induced the transformation of TiO_2_ to Eu_2_Ti_2_O_7_. Meanwhile, the time-resolved fluorescence spectra showed that the average lifetimes of Eu^3+^ ions in TiO_2_ hollow shells were 80.32, 106.54, 193.69, and 131.27 μs at 600, 700, 800°C, and 900°C, respectively (Supplementary Figure S7D). Among them, the TiO_2_:Eu^3+^ hollow shells at 800°C showed the longest average lifetimes, which proved that the increased crystallinity was beneficial to suppress the nonradiative recombination and thus improved the luminescence efficiency of Eu^3+^ ions. In summary, the TiO_2_:Eu^3+^ hollow shells annealed at 800°C exhibited the best crystallinity and fluorescence emission and were used as the source of red light emission in WLED.

### 3.4 Optical properties of CsPb(Cl/Br)_3_/TiO_2_:Eu^3+^ composites

**FIGURE 3:**
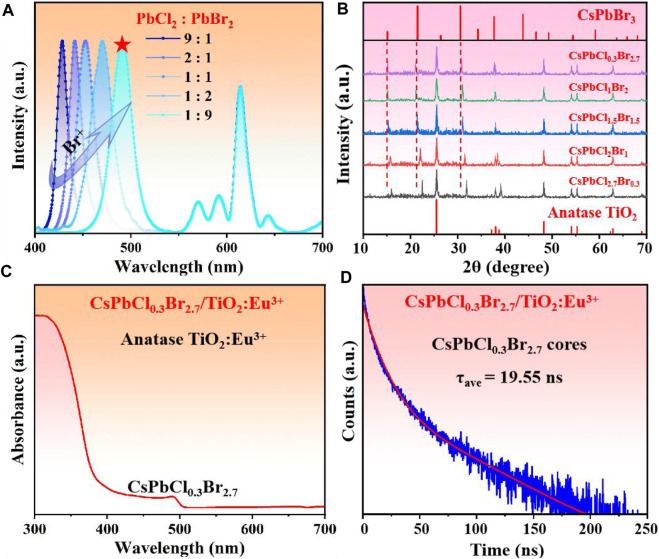
**(A)** PL spectra (λ_em_ = 365 nm) and **(B)** XRD patterns of CsPb(Cl/Br)_3_/TiO_2_:Eu^3+^ composites with different Cl/Br ratios. **(C)** Absorption spectra and **(D)** time-resolved fluorescence spectra of CsPbCl_0.3_Br_2.7_/TiO_2_:Eu^3+^ composites (monitoring peak at 491 nm). The Eu^3+^ ion doping concentration of all samples was 0.6 mmol and the annealing temperature was 800°C.

Subsequently, we had in-situ grown CsPb(Cl/Br)_3_ NCs inside TiO_2_ hollow shells to obtain composites with direct white light emission. It has been known that white light composed of multi-color light, which required multiple light sources to cooperate to achieve the emission. First, We synthesized a series of CsPb(Cl/Br)_3_/TiO_2_:Eu^3+^ composites with different Cl/Br ratios to match the red light (∼614 nm) of TiO_2_:Eu^3+^ hollow shells to obtain pure white light. The pictures of the samples showed that the phosphor gradually changed from blue emission to white as the Br^−^ concentration increased (Supplementary Figure S10A). The corresponding PL spectra (Figure 3A) showed that multiple emission peaks were present for all samples, corresponding to the CsPb(Cl/Br)_3_ NCs (before 500 nm) and the Eu^3+^ ions (after 500 nm). Both characteristic peaks of anatase TiO_2_ and CsPb(Cl/Br)_3_ NCs were also present in the XRD patterns of the samples (Figure 3B). Moreover, the diffraction peaks of NCs shifted toward large angles with increasing Cl^−^ ion content, which was attributed to the replacement of larger Br^−^ by Cl^−^ with smaller ionic radius (Protesescu et al., 2015; Bai et al., 2022). Subsequently, the absorption spectrum of CsPbCl_0.3_Br_2.7_/TiO_2_:Eu^3+^ with the best white light effect was tested (Figure 3C), where the absorption peak at 490 nm was assigned to CsPb(Cl/Br)_3_ NCs and the absorption peak before 400 nm to TiO_2_:Eu^3+^ hollow shells. Further, we monitored the fluorescence lifetime of CsPbCl_0.3_Br_2.7_/TiO_2_:Eu^3+^ composites and pure phase CsPbCl_0.3_Br_2.7_ NCs at 491 nm to demonstrate the passivation effect of TiO_2_:Eu^3+^ hollow shells (Figure 3D). The results showed that the average lifetime of CsPb(Cl/Br)_3_/TiO_2_:Eu^3+^ composites was 19.55 ns, while the pure CsPbCl_0.3_Br_2.7_ NCs was only 10.52 ns (Supplementary Figure S10B). The significant extension of the lifetime of NCs proved that the TiO_2_:Eu^3+^ shell layer can effectively isolate the PL quenching molecules from entering the interior and avoided the increase in the density of defect states on the surface of NCs by the external environment, thus improving the radiative recombination efficiency (Ravi et al., 2020; Ji et al., 2021). Finally, we compared the stability of the composites and pure phase CsPbCl_0.3_Br_2.7_ NCs, and the results were shown in Supplementary Figure S11. First, after 5 h of immersion in water, the CsPbCl_0.3_Br_2.7_/TiO_2_:Eu^3+^ could retain 72% of the fluorescence intensity, and the CsPbCl_0.3_Br_2.7_ NCs were 55% (Supplementary Figures S11A, B). The fluorescent intensity of both materials was dramatically reduced, attributed to external water molecules that could diffuse to the interior through the surface pores of the hollow shells, resulting in no appreciable improvement in water stability. However, the test results of thermal stability were surprising. After continuous heating at 80°C, the composites could still maintain more than 90% of PL intensity, while the CsPbCl_0.3_Br_2.7_ NCs had been mostly quenched (Supplementary Figures S11C, D). The light stability also exhibited similar results, demonstrating that the TiO_2_ shells can isolate some of the PL quenching molecules, allowing the perovskite NCs to maintain the stability of the crystal structure while withstanding more severe environmental aggression (Supplementary Figures S11E, F). Therefore, all the above results demonstrated that the CsPbCl_0.3_Br_2.7_/TiO_2_:Eu^3+^ composites had efficient white light emission and excellent stability.

### 3.5 Preparation and performance of WLEDs

**FIGURE 4:**
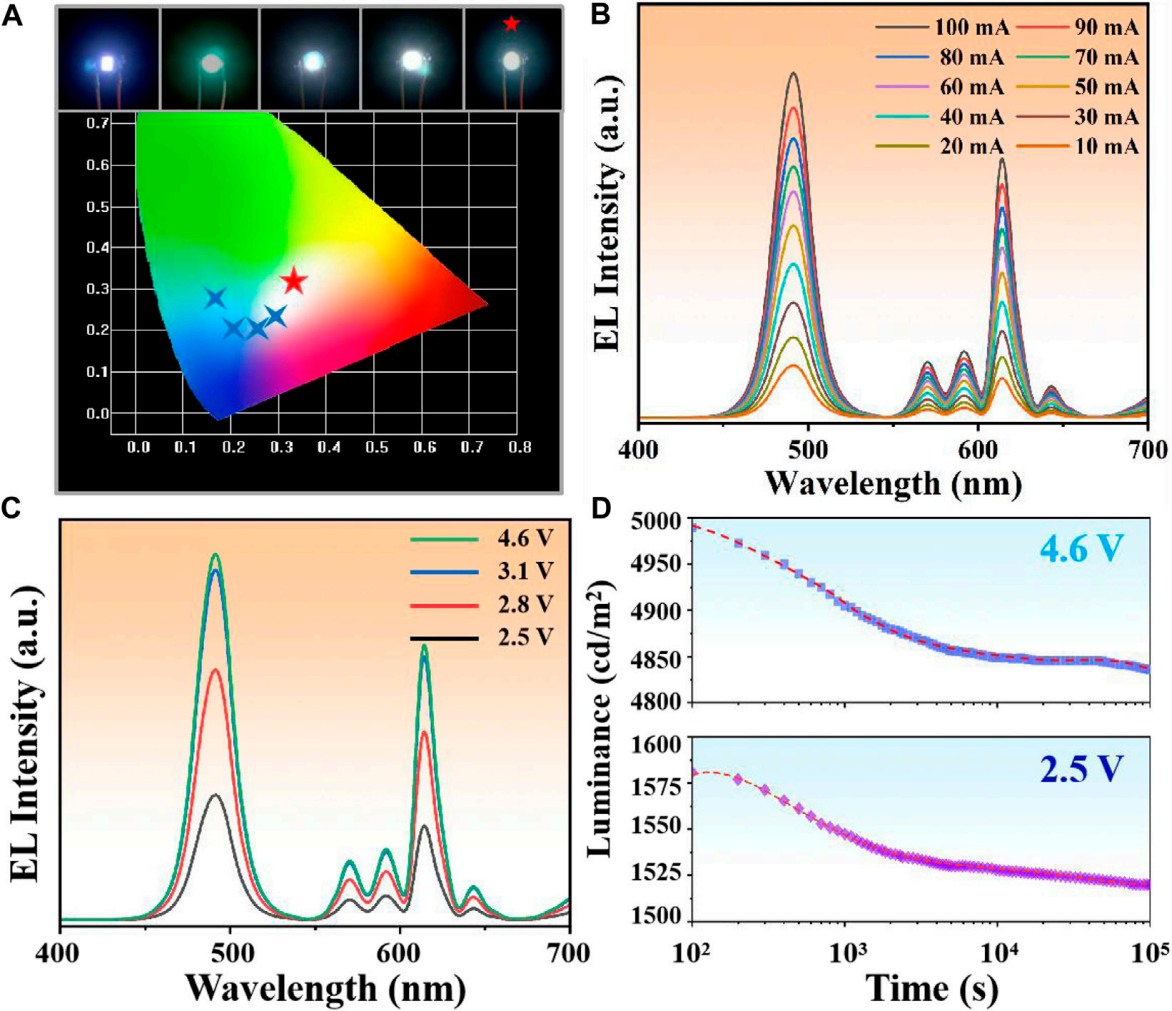
**(A)** Chromaticity coordinates of LEDs prepared from composites with different Cl/Br ratios. Among them, CsPbCl_0.3_Br_2.7_/TiO_2_:Eu^3+^ WLED showed the purest white light emission (0.318, 0.326). **(B)** EL spectra of CsPbCl_0.3_Br_2.7_/TiO_2_:Eu^3+^ WLED at 10–100 mA drive current. **(C)** EL spectra of CsPbCl_0.3_Br_2.7_/TiO_2_:Eu^3+^ WLED at different drive voltages. **(D)** Stability fitting curves of CsPbCl_0.3_Br_2.7_/TiO_2_:Eu^3+^ WLED at 2.5 V and 4.6 V.

As one of the most important applications of CsPbX_3_ NCs, the luminous efficiency and stability of LED determined the development prospect of the materials (Dong et al., 2020; Yoon et al., 2023). We prepared a series of LEDs by encapsulating composites with polymers (Polystyrene) and integrating them onto 365 nm chips. Among them, the CsPbCl_0.3_Br_2.7_/TiO_2_:Eu^3+^ LED exhibited the purest white light emission (0.318, 0.326) (Figure 4A), which was consistent with the observation of the previous samples. Further, the EL spectra at different drive currents (Figure 4B) and different drive voltages (Figure 4C) showed that the WLED peak positions were not shifted, demonstrating the good luminous stability of the device. The time-dependent EL decay curve of CsPbCl_0.3_Br_2.7_/TiO_2_:Eu^3+^ WLED (Figure 4D) showed that the luminous intensity of WLED had a fast decaying trend (decaying about 2–3%) in the first 1,000 s at either high voltage (4.6 V) or low voltage (2.5 V), and then tends to level off. This phenomenon was attributed to the rapid heating of the device surface, which caused thermal decomposition of the NCs unshielded by the TiO_2_ shells. However, after reaching thermal equilibrium (>1,000 s), the decay trend slows down by two orders of magnitude, demonstrating that the TiO_2_ shells isolated the external thermal environment, and effectively decelerated the decomposition rate of NCs.

**FIGURE 5:**
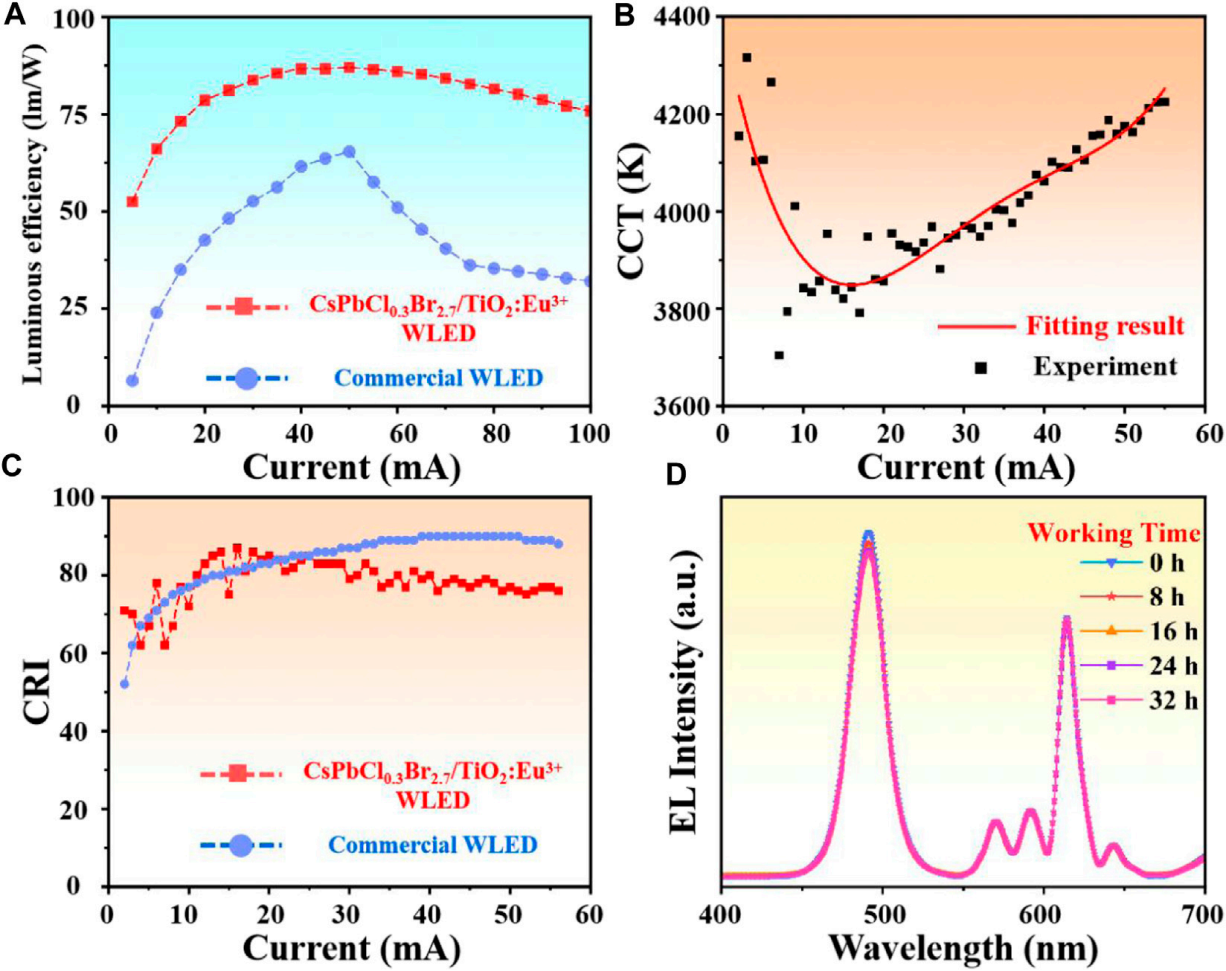
**(A)** Comparison of the luminous efficiency of CsPbCl_0.3_Br_2.7_/TiO_2_:Eu^3+^ WLED and commercial WLED. **(B)** The correlated color temperatures of CsPbCl_0.3_Br_2.7_/TiO_2_:Eu^3+^ WLED at 2–60 mA drive current. **(C)** Comparison of the color rendering index of CsPbCl_0.3_Br_2.7_/TiO_2_:Eu^3+^ WLED and commercial WLED. **(D)** EL spectra of CsPbCl_0.3_Br_2.7_/TiO_2_:Eu^3+^ WLED operating continuously for 32 h at 20 mA drive current.

Subsequently, the highest luminous efficiency of CsPbCl_0.3_Br_2.7_/TiO_2_:Eu^3+^ WLED was 87.39 lm/W, which was approximately 38.4% higher than commercial WLED (Maximum about 63.14 lm/W) (Figure 5A). Such high luminescence efficiency was attributed to the effective passivation of NCs surface defects by the external highly crystalline TiO_2_:Eu^3+^ hollow shells. Moreover, the correlated color temperature (CCT) fluctuated from 3,700 to 4,300 K with a warm white color, which can effectively avoid the damage of blue light to human eyes (Figure 5B) (Fan et al., 2023; He et al., 2023; Yang et al., 2023). Figure 5C exhibited the color rendering index (CRI) comparison between CsPbCl_0.3_Br_2.7_/TiO_2_:Eu^3+^ WLED and commercial WLED. The results indicated that the CRI of CsPbCl_0.3_Br_2.7_/TiO_2_:Eu^3+^ WLED remained above 60 at all current conditions and achieved the maximum of 87 at 16 mA drive current, which was close to the commercial LED (maximum 90). Finally, the luminous intensity test under long time operation also showed that the WLED had practical application prospects. Even after 32 h of continuous operation, the luminous intensity remained above 95%, and the peak position was not significantly shifted (Figure 5D). In conclusion, the passivation strategy using highly crystalline TiO2:Eu3+ hollow shells as protective layer and emission source can expand the application of perovskite materials in optoelectronic field.

## 4 Conclusion

In summary, CsPbCl_0.3_Br_2.7_/TiO_2_:Eu^3+^ composites with multi-color light emission were in-situ grown with TiO2 as the protective shell and Eu3+ ions as the red light source. It was found that TiO_2_:Eu^3+^ hollow shells had the best red light emission and crystallinity when the Eu^3+^ doping amount was at 0.6 mmol and the annealing temperature was 800°C. Meanwhile, when the Cl^−^/Br^−^ ratio of CsPb(Cl/Br)_3_ NCs was 1:9, the emission peaks of NCs and Eu^3+^ had the best matching effect, resulting in pure white light emission. The stability of the CsPbCl_0.3_Br_2.7_/TiO_2_:Eu^3+^ composites was significantly improved due to the protection of the external highly crystalline TiO_2_:Eu^3+^ shell layer. Moreover, the WLED prepared with the materials exhibited a luminous efficiency of 87.39 lm/W and long-time operating stability. In conclusion, the highly efficient and stable white light emission by packaging CsPb(Cl/Br)_3_ NCs in high crystallinity shells showed great potential in the field of optoelectronics.

## Data Availability

The original contributions presented in the study are included in the article/Supplementary Material, further inquiries can be directed to the corresponding author.
