# Supplementary material for: Preparation of CsPb(Cl/Br)3/TiO2:Eu3+ composites for white light emitting diodes
Source: Front Chem. 2023 May 19;11:1199863. doi: 10.3389/fchem.2023.1199863 (PMC10235637; doi:10.3389/fchem.2023.1199863)
Supplement: Supplementary file 1 [file DataSheet1.docx]

Supplementary Material

**Preparation of CsPb(Cl/Br)_3_/TiO_2_:Eu^3+^ Composites for White Light Emitting Diodes**

**Chen Zhang^1^, Minqiang Wang^1*^, Jindou Shi^1^, Junnan Wang^1^, Zheyuan Da^1^, Yun Zhou^1^, Youlong Xu^1^, Nikolai V. Gaponenko^2^ and Arshad Saleem Bhatti^3^**

***Correspondence:** Minqiang Wang: [mqwang@xjtu.edu.cn](mailto:mqwang@xjtu.edu.cn)

**
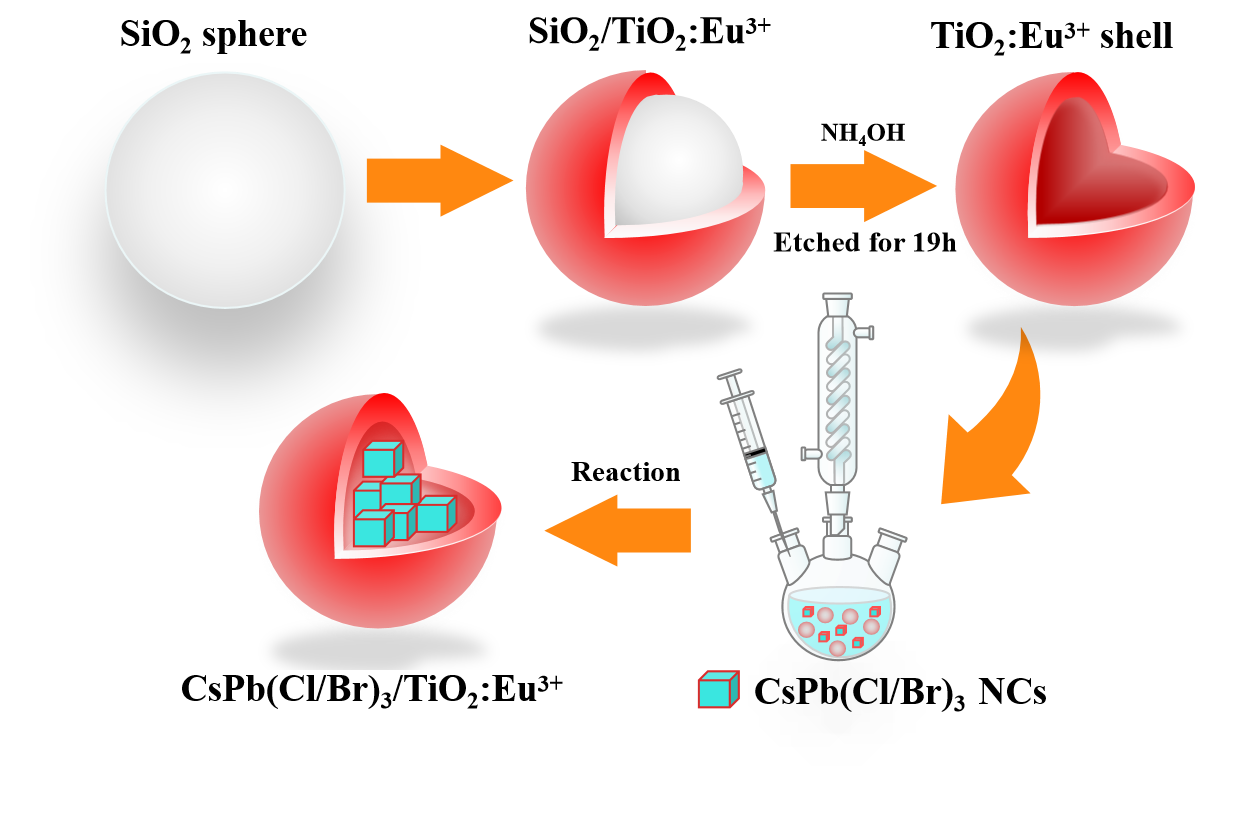
Figure S1.** Flow chart for the preparation of CsPb(Cl/Br)_3_/TiO_2_:Eu^3+^ composites.

The process of preparation of CsPb(Cl/Br)_3_/TiO_2_:Eu^3+^ composites was divided into the following main steps. First, Eu^3+^ ion-doped TiO_2_ hollow shells were prepared. SiO_2_ spheres were used as hard templates, and TiO_2_ particles were encapsulated on SiO_2_ spheres by the hydrolysis reaction of TBOT and doped with 0.2~1.2 mmol of Eu(NO_3_)_3_. After high temperature annealing and NH_4_OH etching, TiO_2_:Eu^3+^ hollow shells with bright down-conversion red light emission can be prepared. In the second step, the TiO_2_:Eu^3+^ hollow shells were placed into the precursor solution mixed with PbCl_2_ and PbBr_2_, and the NCs were crystallized inside the TiO_2_ hollow shells by the thermal injection method to obtain the CsPb(Cl/Br)_3_/TiO_2_:Eu^3+^ composites.

**
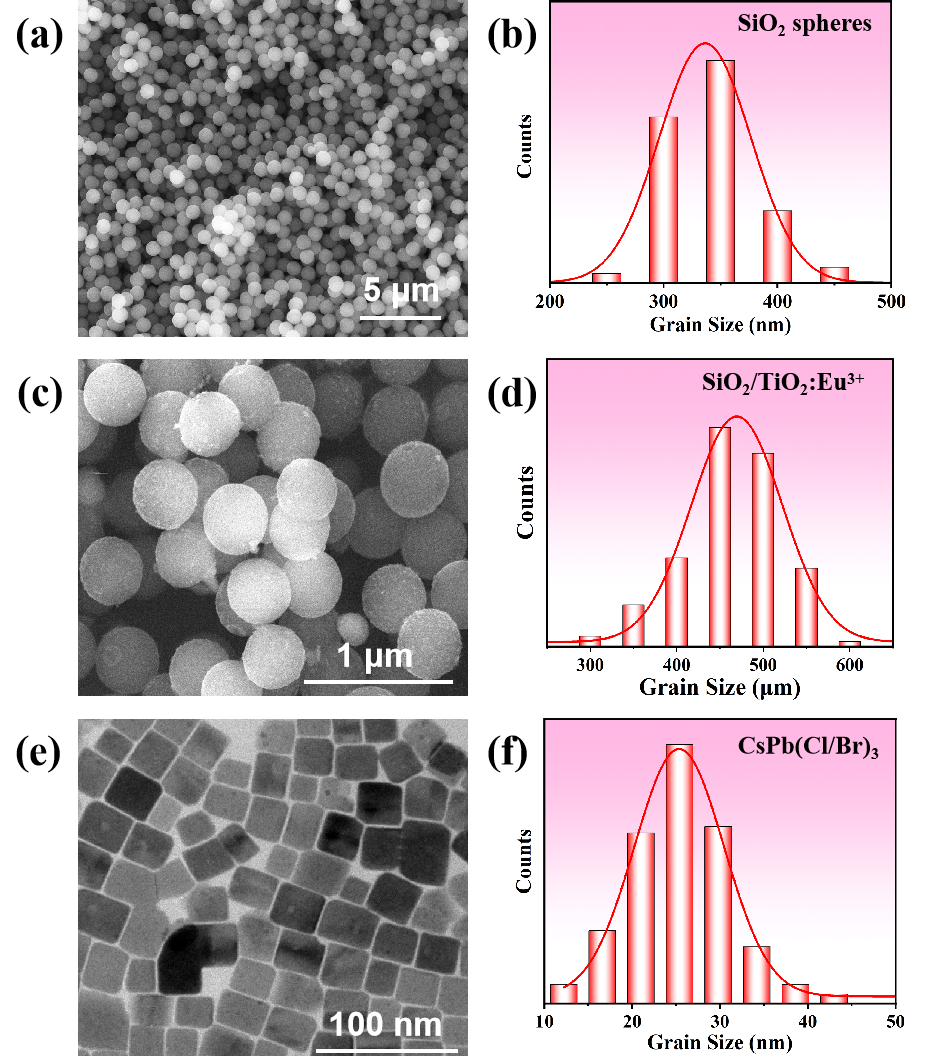
Figure S2.** SEM images and size distribution of (a, b) SiO_2_ spheres, (c, d) SiO_2_/TiO_2_:Eu^3+^ composites and (e, f) CsPb(Cl/Br)_3_ NCs.

**
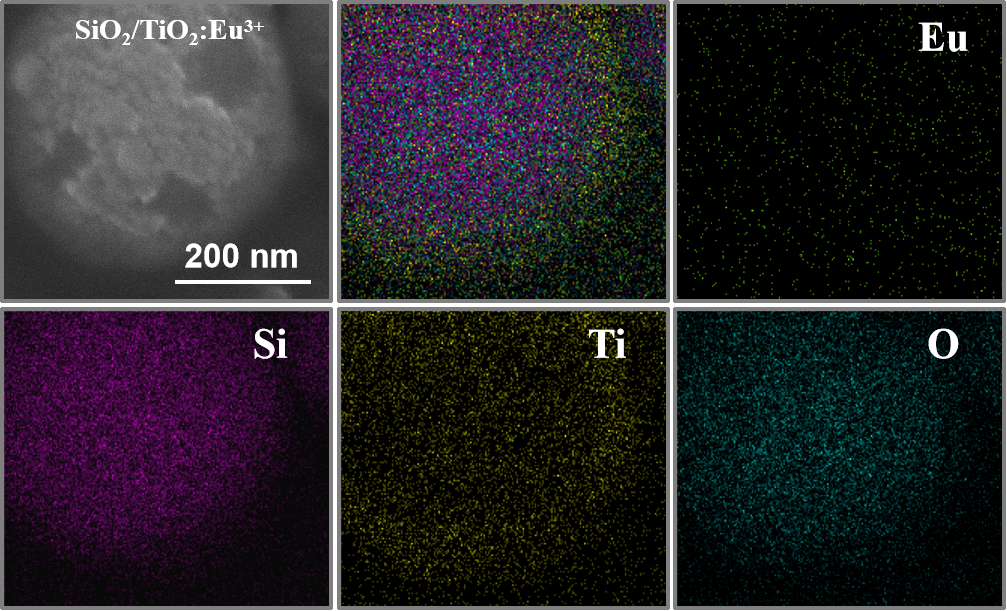
Figure S3.** EDS mapping of SiO_2_/TiO_2_:Eu^3+^ composites.**
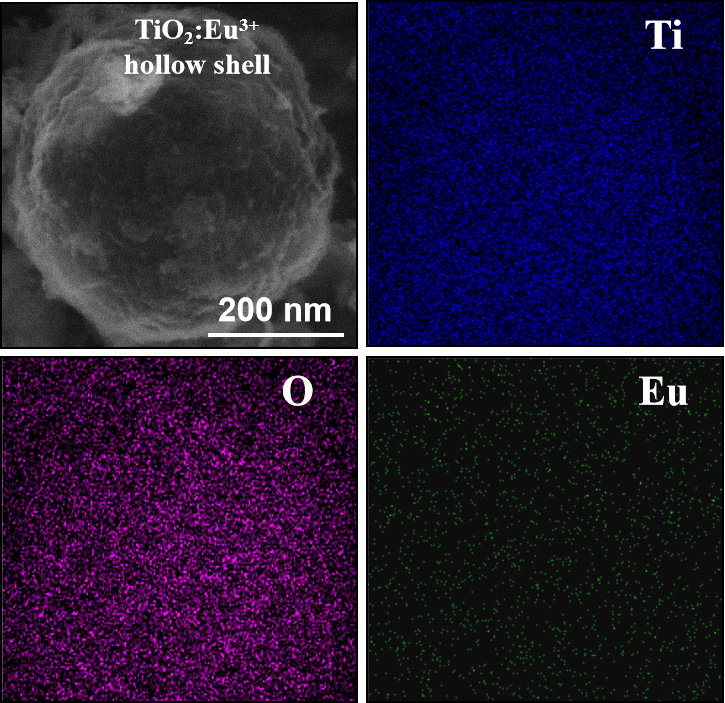
Figure S4.** EDS mapping of TiO_2_:Eu^3+^ hollow shells.

**Figure
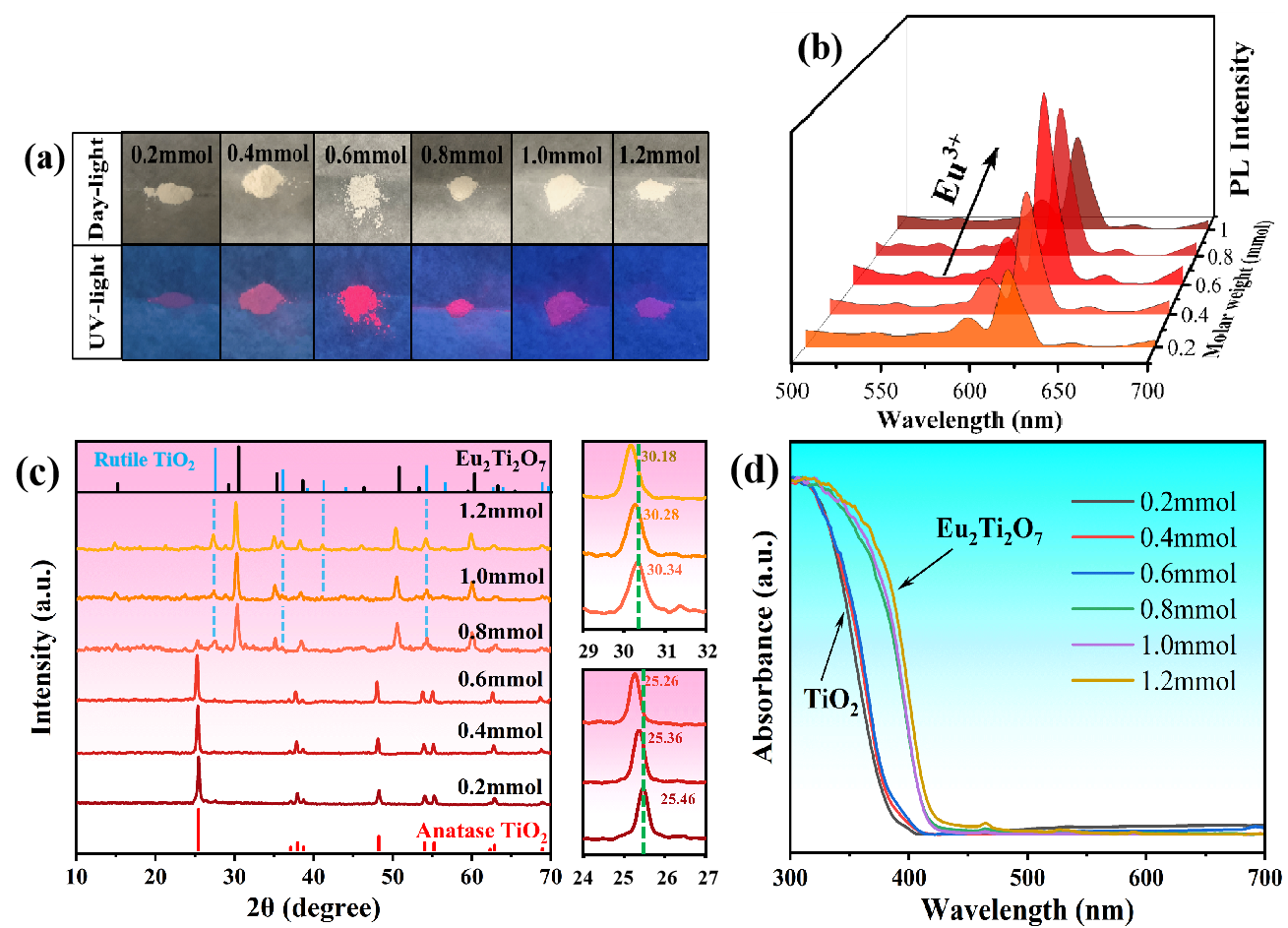
 S5.** (a) Sample pictures of 0.2~1.2 mmol Eu^3+^ doped TiO_2_ hollow shells under day-light and 365 nm UV-light. (b) PL spectra (λ_em_=365 nm), (c) XRD patterns and (d) UV-Vis absorption spectra of 0.2~1.2 mmol Eu^3+^ doped TiO_2_ hollow shells. The samples of this part were annealed at 800 ℃.

**
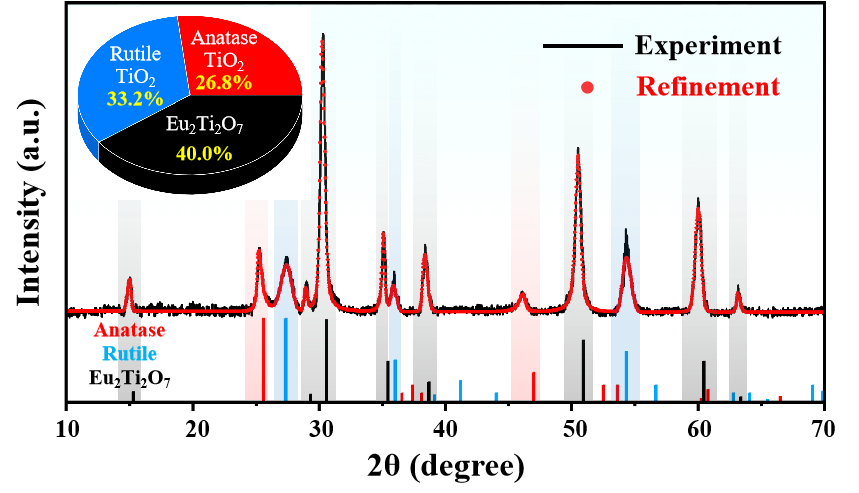
Figure S6.** XRD patterns of TiO_2_ hollow shells under 0.8 mmol Eu^3+^ doping. The black line is the XRD pattern of the product; the red dots are the Rietveld refinement. The inset exhibits the calculated content of the ternary phase.

**
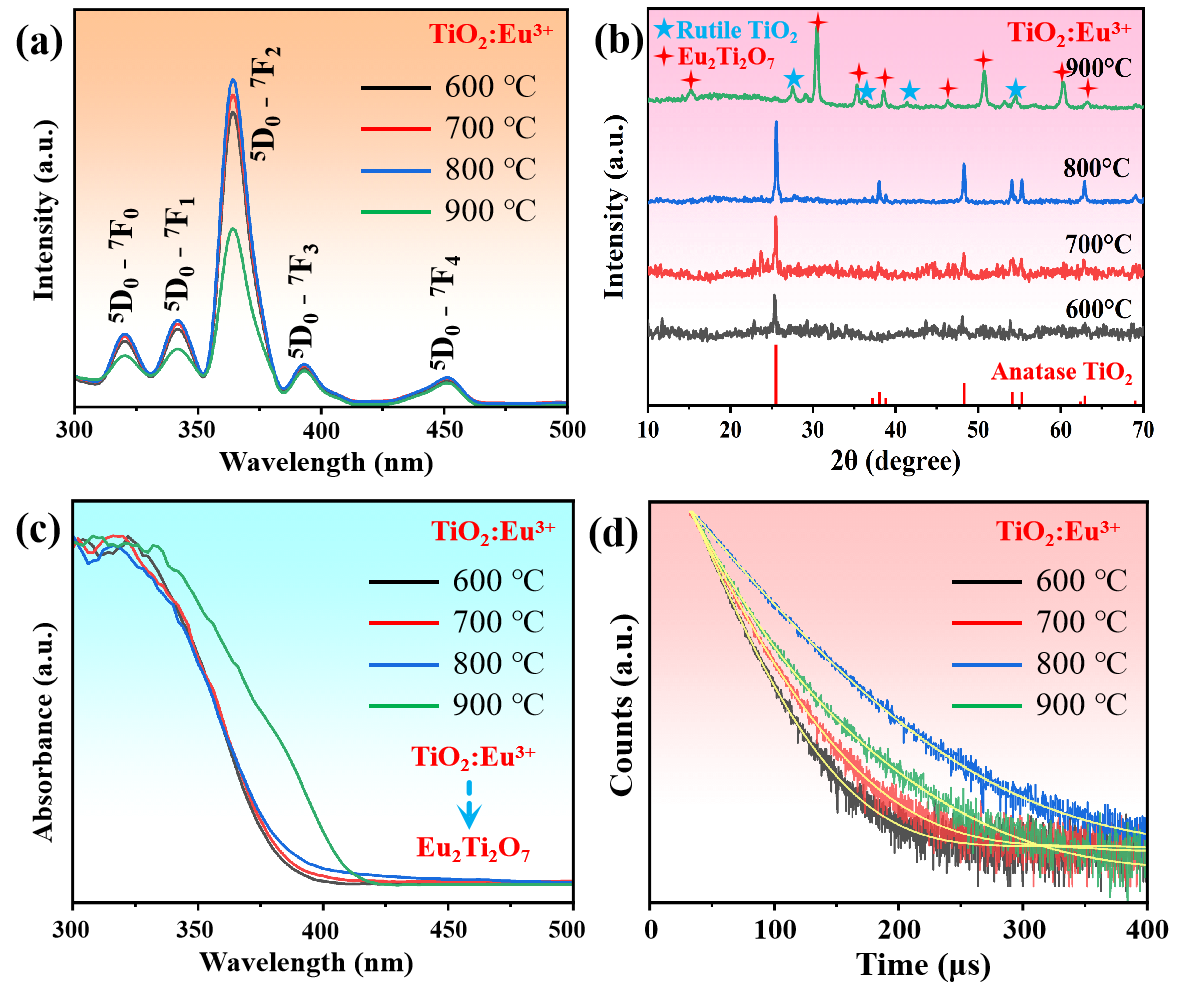
Figure S7.** (a) PL spectra (λ_em_=365 nm), (b) XRD patterns, (c) UV-Vis absorption spectra and (d) time-resolved fluorescence spectra (monitoring peak at 614 nm) of 0.6 mmol Eu^3+^ doped TiO_2_ hollow shells at 600~900 ℃. The Eu^3+^ ion doping concentration of all samples was 0.6 mmol.

**
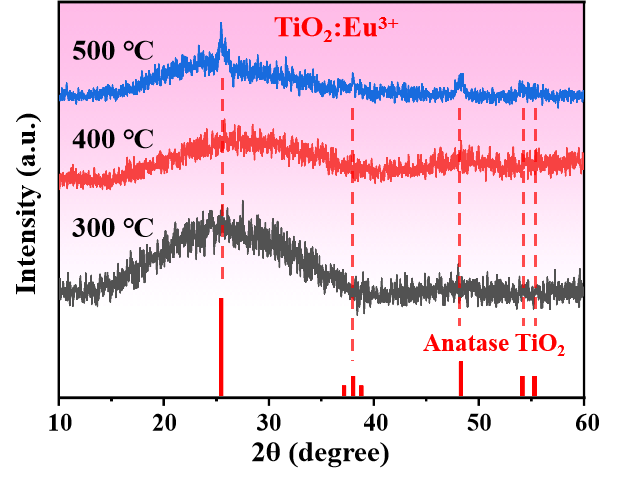
Figure S8.** XRD patterns of TiO_2_:Eu^3+^ shells at annealing temperatures of 300, 400 and 500 ℃.

**Figure S
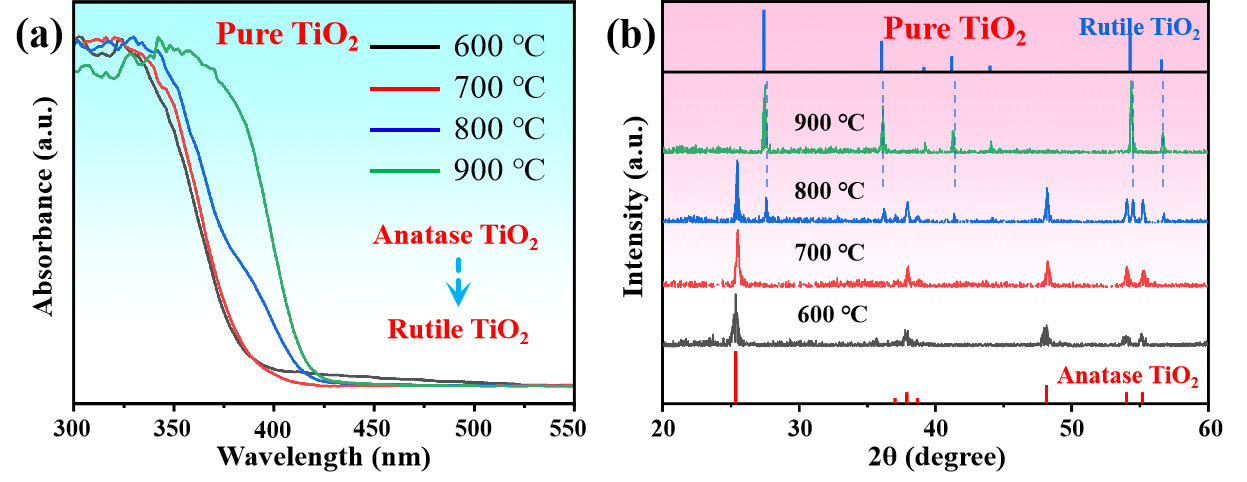
9.** After annealing at 600~900 ℃ for 3 h, the (a) UV-Vis absorption spectra and (b) XRD patterns of pure TiO_2_.

**
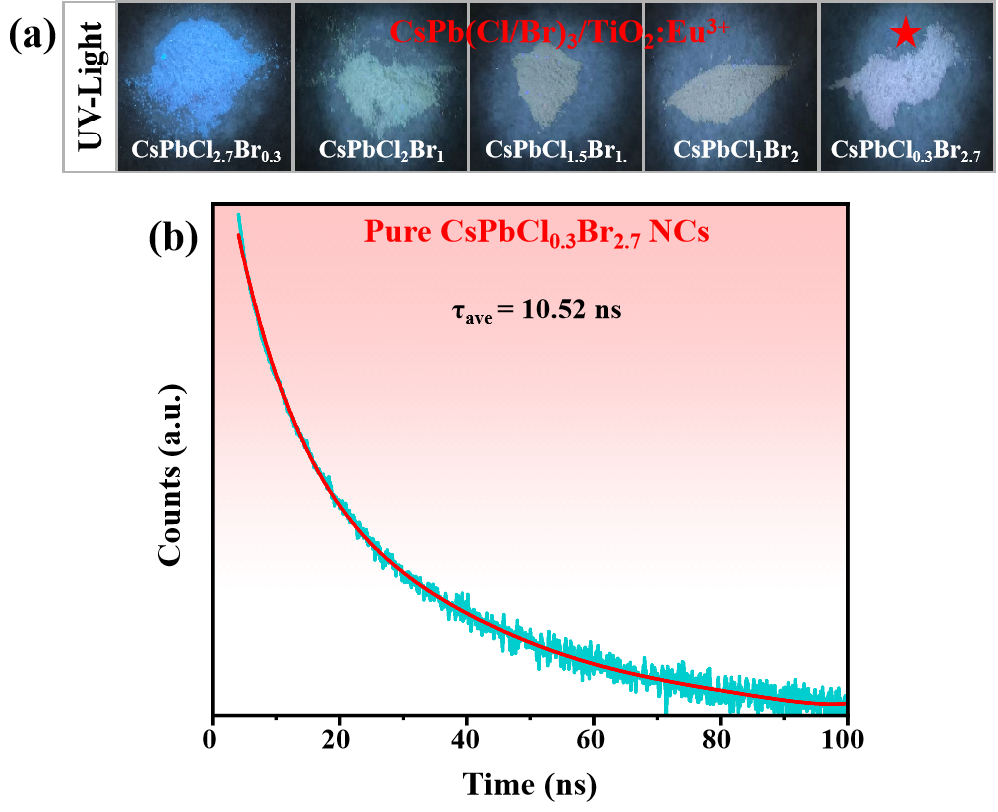
Figure S10.** (a) Pictures of CsPb(Cl/Br)_3_/TiO_2_:Eu^3+^ composites with different Cl/Br rates under 365 nm UV-light. (b) Time-resolved PL spectra and fitting curves of pure CsPbCl_0.3_Br_2.7_ NCs (monitoring peak at 614 nm).**
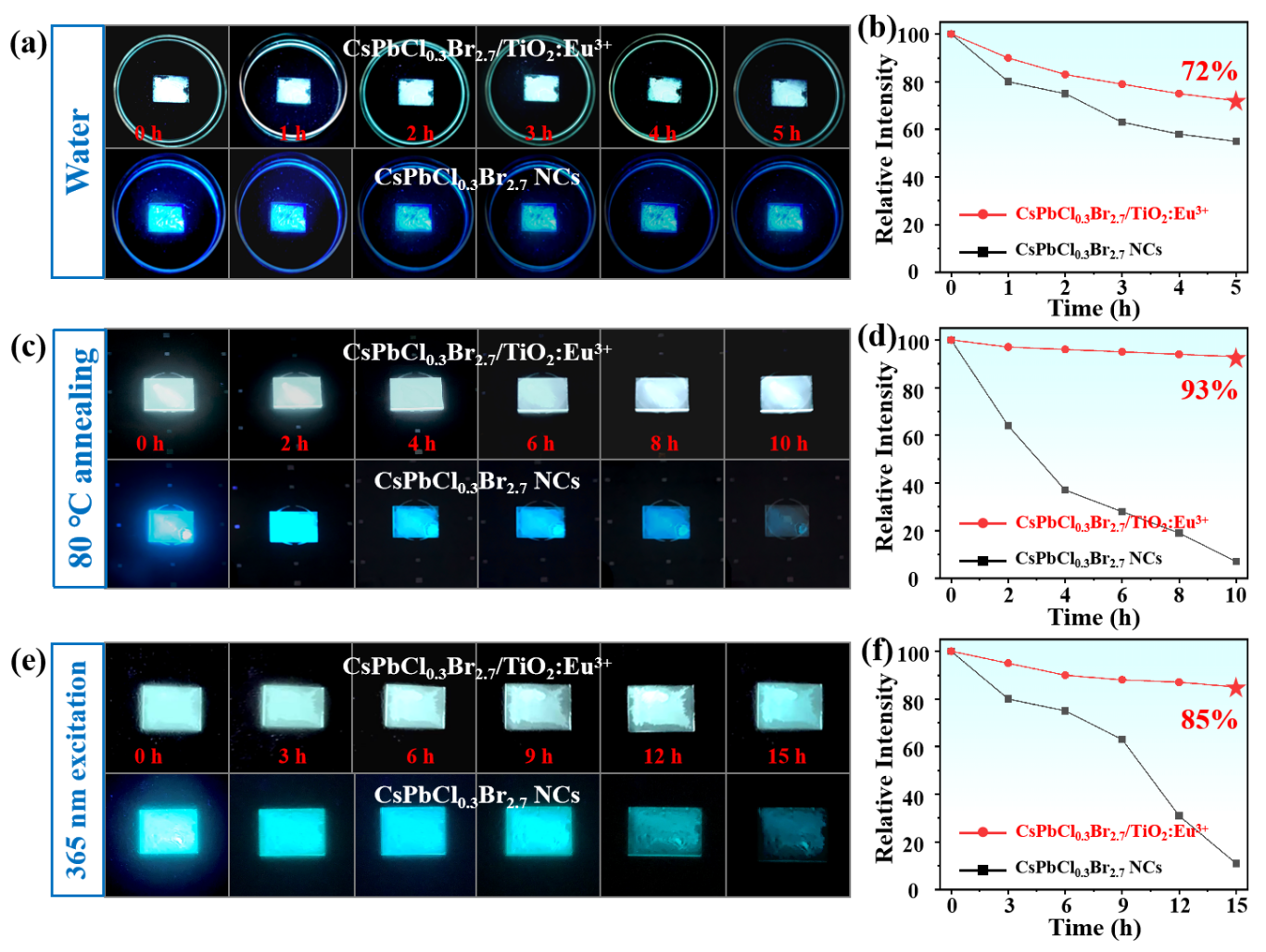
Figure S11.** Comparison of stability between CsPbCl_0.3_Br_2.7_/TiO_2_:Eu^3+^ composites and pure CsPbCl_0.3_Br_2.7_ NCs. (a) Sample photos of two materials soaked in water for 5 h. (b) Relative PL intensity at different soaking times. (c) Sample photos of two materials after heat treatment at 80 ℃ for 10 h. (d) Relative PL intensity at different heating times. (e) Sample photos of two materials irradiated with 365 nm UV-light for 15 h. (f) Relative PL intensity at different light times. Relative intensity was defined as *I/I_0_* × 100%. Where *I* was the tested PL intensity and *I_0_* was the initial PL intensity.
